# Fever and Ague

**Published:** 1871-02

**Authors:** 


### FEVER AND AGUE

Is said to be cured by dissolving a heaping tea-spoon of common salt in half a glass of water, and drinking it on rising, three mornings in succession. It would not be wise to call this ridiculous until thoroughly tried. An old negro once said that he knew a tree, the bark of which would cure chill and fever; the people tried it, and, finding that it never failed in that country, gave the man a large amount of money to show the tree; from this bark quinine is now made.

If common table-salt can cure fever and ague, it may be added to the increasing list of "Food Cures," for salt is on all our tables. Still it would be better to have no fever and ague, by draining all ponds within five miles of the locality where it prevails, in the winter; then either fill them up or plough the bottoms and cultivate them, thus obtaining a large amount of healthful food from spots which caused disease and death to make their ravages through whole neighborhoods.

